# Molecular Cloning and Pharmacological Properties of an Acidic PLA_2_ from *Bothrops pauloensis* Snake Venom

**DOI:** 10.3390/toxins5122403

**Published:** 2013-12-04

**Authors:** Francis Barbosa Ferreira, Mário Sérgio Rocha Gomes, Dayane Lorena Naves de Souza, Sarah Natalie Cirilo Gimenes, Letícia Eulalio Castanheira, Márcia Helena Borges, Renata Santos Rodrigues, Kelly Aparecida Geraldo Yoneyama, Maria Inês Homsi Brandeburgo, Veridiana M. Rodrigues

**Affiliations:** 1 Institute of Genetics and Biochemistry, Federal University of Uberlândia, UFU, 38400902 Uberlândia-MG, Brazil; E-Mails: francisbbio@yahoo.com (F.B.F.); mrochauesb@yahoo.com.br (M.S.R.G.); dayane_lorena@yahoo.com.br (D.L.N.S.); sarah_gi_menes@hotmail.com (S.N.C.G.); leticiacasth@yahoo.com.br (L.E.C.); rsrodrigues@ingeb.ufu.br (R.S.R.); kellyagy@gmail.com (K.A.G.Y.); homsi@ufu.br (M.I.H.B.); 2 Department of Chemical and Physical, State University of Southwest Bahia (UESB), 45506-210 Jequié-BA, Brazil; 3 Ezequiel Dias Foundation, FUNED, 30510-010 Belo Horizonte-MG, Brazil; E-Mail: mhborgesb@gmail.com

**Keywords:** *Bothrops pauloensis*, phospholipase A_2_, molecular cloning

## Abstract

In this work, we describe the molecular cloning and pharmacological properties of an acidic phospholipase A_2_ (PLA_2_) isolated from *Bothrops pauloensis* snake venom*.* This enzyme, denominated BpPLA_2_-TXI, was purified by four chromatographic steps and represents 2.4% of the total snake venom protein content. BpPLA_2_-TXI is a monomeric protein with a molecular mass of 13.6 kDa, as demonstrated by Matrix-assisted laser desorption/ionization time-of-flight mass spectrometry (MALDI-TOF) analysis and its theoretical isoelectric point was 4.98. BpPLA_2_-TXI was catalytically active and showed some pharmacological effects such as inhibition of platelet aggregation induced by collagen or ADP and also induced edema and myotoxicity. BpPLA_2_-TXI displayed low cytotoxicity on TG-180 (CCRF S 180 II) and Ovarian Carcinoma (OVCAR-3), whereas no cytotoxicity was found in regard to MEF (Mouse Embryonic Fibroblast) and Sarcoma 180 (TIB-66). The N-terminal sequence of forty-eight amino acid residues was determined by Edman degradation. In addition, the complete primary structure of 122 amino acids was deduced by cDNA from the total RNA of the venom gland using specific primers, and it was significantly similar to other acidic D49 PLA_2_s. The phylogenetic analyses showed that BpPLA_2_-TXI forms a group with other acidic D49 PLA_2_s from the gender *Bothrops*, which are characterized by a catalytic activity associated with anti-platelet effects.

## 1. Introduction

*Bothrops pauloensis* is a venomous snake widely distributed throughout the Brazilian territory, except in the Amazonian region of Brazil. This species is particularly common in the Triangulo Mineiro region, in the southwest of Minas Gerais State [1].

Rodrigues *et al.* [2] described the repertoire of venom toxins from *B. pauloensis* by snake proteomics and venom gland transcriptomic surveys. The main toxins present in *B. pauloensis* snake venom include metalloproteinases, phospholipases A_2_, serine proteinases, l-amino acid oxidases, disintegrins, nucleotidases and hyaluronidases, among others [2]. Both approaches indicated metalloproteinases, vasoactive (bradykinin-potentiating) peptides and phospholipases A_2_ as the major toxin classes. 

Phospholipases A_2_ (PLA_2_) (E.C. 3.1.1.4) represent a superfamily of lipolytic enzymes that catalyze the hydrolysis of the 2-acyl ester of the phospholipids, releasing free fatty acids and lysophosphatids [3,4]. PLA_2_s are classified into 15 groups that are further subdivided into several groups (sPLA_2_—secreted; cPLA_2_—cytosolic; iPLA_2_—Ca^2+^ independent; LpPLA_2_—lipoprotein-associated), all of which display differences in amino acid sequence, disulfide bonds, tissue specificity and cellular functions [5,6]. 

PLA_2_s are present in snake venoms and are characterized by low molecular mass (13–18 kDa), histidine at the catalytic site, Ca^2+^ bound at the active site, as well as six conserved disulfide bonds with one or two variable disulfide bonds [7]. These PLA_2_s can be divided into two groups, I and II, whereby the second is subdivided into two classes, and are present in snake venoms from the Viperidae family. These classes are D49 PLA_2_s, which display an Asp residue at position 49, with high catalytic activity upon artificial substrates; and Lys49 PLA_2_s, that presents a Lys residue at position 49, with low or no catalytic activity [8,9].

The different PLA_2_s isoforms that have already been isolated from *B. pauloensis* snake venom include: BnpTX-I and BpnTX-II (D-49 basic) [10], BnSP-6 and BnSP-7 (K-49 basic) [11] and Bp-PLA_2_ (D-49 acidic) [12]. These toxins present some toxic and/or pharmacological effects characterized as neurotoxicity, myotoxicity, cytotoxicity, inhibition of platelet aggregation, anticoagulation, edema, convulsion and hypotension [3,8,9]. The present study describes the molecular cloning, as well as the enzymatic and pharmacological properties of an acidic phospholipase A_2_ from *B. pauloensis* snake venom.

## 2. Results and Discussion

**Figure 1:**
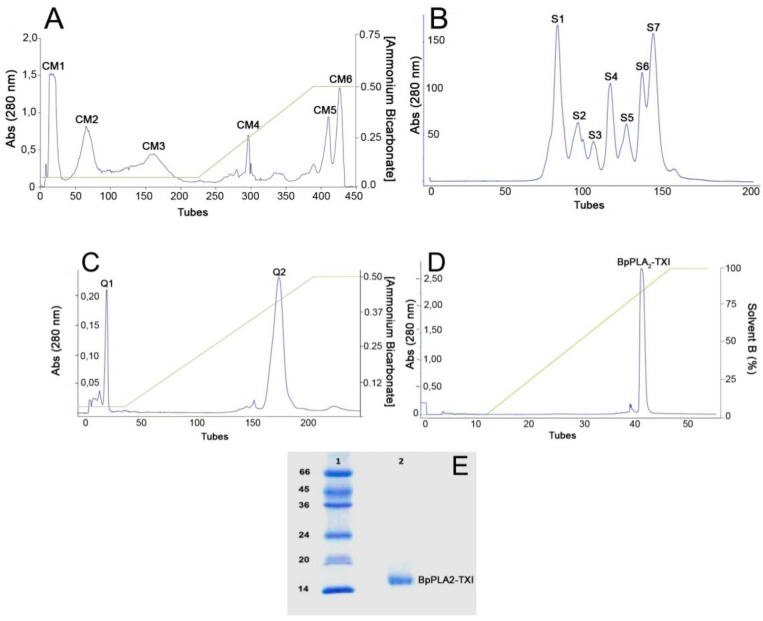
Sequential purification steps of BpPLA_2_-TXI. (**A**) *Bothrops pauloensis* venom (170 mg) on CM-Sepharose Fast Flow previously equilibrated with ammonium bicarbonate (AMBIC) buffer 0.05M pH 7.8. A gradient was then applied up to 0.5 M (AMBIC) buffer, pH 7.8. Fractions of 1 mL/tube were collected in a 6 mL/h flow rate at room temperature; (**B**) CM1 (60 mg) on Sephacryl-S300 previously equilibrated and eluted with AMBIC buffer (0.05 M; pH 7.8) at a flow rate of 12.0 mL/h and fractions of 2.0 mL/tube were collected; (**C**) S4 was rechromatographed on HiTrap Q FF column, equilibrated with AMBIC (0.05 M; pH 7.8). A gradient was then applied up to the column using AMBIC buffer (0.5 M, pH 7.8) at flow rate of 6.0 mL/h and fractions of 1 mL/tube were collected; (**D**) The active fraction (Q2) on reverse phase-high performance liquid chromatography (RP-HPLC) C2–C18 and PLA_2_ was eluted using Solvent A (0.1% TFA, 4% acetonitrile) to 100% of the Solvent B (0.1% TFA, 80% acetonitrile) at the flow rate of 0.5 mL/min for 33 min and fractions of 0.5 mL/tube; (**E**) sodium dodecyl sulfate polyacrylamide gel electrophoresis (SDS-PAGE) at 12% (*w*/*v*). Line 1: molecular mass markers; Line 2: acidic PLA_2_ (BpPLA_2_-TXI).

**Figure 2:**
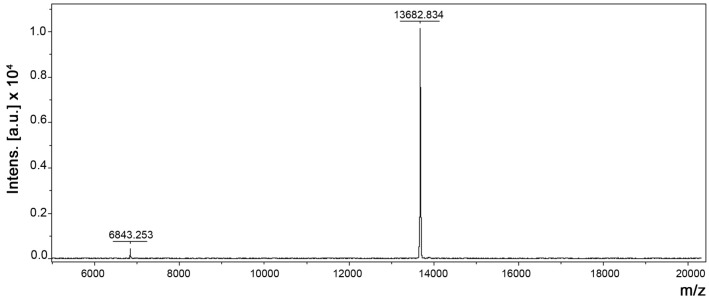
Molecular mass determination of BpPLA_2_-TXI by Matrix-assisted laser desorption/ionization time-of-flight mass spectrometry (MALDI-TOF) (Bruker Daltonics, Bremen, Germany).

An acidic PLA_2_ isolated from the venom of *B. pauloensis* was obtained by four chromatographic procedures in the present work. The first step consisted of an ion exchange chromatography on CM-Sepharose column and resulted in six major protein peaks (Figure 1A). The fraction named CM-1, with phospholipase A_2_ activity (Table 1), was further fractionated on Sephacryl-S300 HR HiPrep 26/60 (filtration chromatography) and resulted in seven new protein fractions, denominated S1–S7 (Figure 1B). S4, which displayed high PLA_2_ activity, was applied on a Hi-Trap Q FF ion-exchange column and resulted in two peaks, Q1 and Q2 (Figure 1C). The Q2 fraction was then applied on a reverse phase high performance liquid chromatography (HPLC) C2–C18 µRPC 4.6/100 (GE HealthCare) and the acidic PLA_2_, named BpPLA_2_-TXI, was eluted with approximately 80% of the Solvent B (Figure 1D). BpPLA_2_-TXI was shown to be homogeneous by sodium dodecyl sulfate polyacrylamide gel electrophoresis (SDS-PAGE) (Figure 1E) with an apparent molecular mass of 15,000 Da in the presence of the reducing agent. Analysis by matrix-assisted laser desorption/ionization time-of-flight mass spectrometry (MALDI-TOF) (Figure 2) of the intact protein revealed a purified protein with a molecular mass of 13,682 Da. The theoretical *pI* of BpPLA_2_-TXI was found to be 4.98, as calculated based on a deduced sequence of cDNA by using the program CLC Sequence Viewer 6 (CLC bio, Aarhus, Denmark; http://www.clcbio.com/index.php?id=28), confirming that BpPLA_2_-TXI is an acidic PLA_2_.

**Table 1:** Protein yield and recovery of enzymatic activity of the crude venom and fractions.

Fraction	Protein *		Phospholipase activity **		
	mg	% Rec	U/mg	U-total	Purification factor
Crude Venom	170	100	51	8670	1.0
CM1	60	35	87	5220	1.7
S4	20	11.7	113	2260	2.2
Q2	7.5	4.4	135	1012	2.6
BpPLA_2_-TXI	4.5	2.6	142	639	2.78

* Protein concentration was determined by Bradford (1976); ** Phospholipase A_2_ activity was determined by DE HAAS (1968).

Table 1 shows total protein yield and recovery of PLA_2_ activity. BpPLA_2_-TXI exhibited a specific PLA_2_ activity of 142 U/mg, three-fold higher than the crude venom (51 U/mg). BpPLA_2_-TXI, an acidic PLA_2_ isoenzyme isolated from *B. pauloensis* venom, presented a considerable recovery of the specific phospholipase activity (Table 1). These results are in agreement with previous studies involving PLA_2_ from snake venoms, in which the catalytic activity of D49 acidic PLA_2_s are higher than that related to D49 basic PLA_2_s [8,9,11,12].

**Figure 3:**
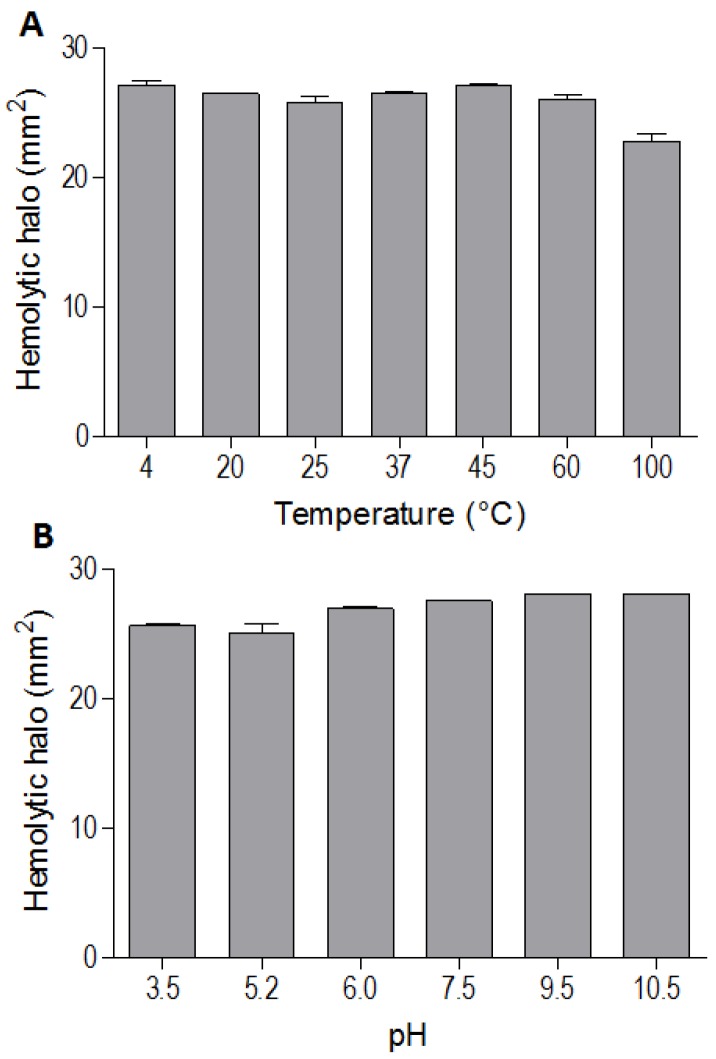
Effects of different pH values and temperatures on phospholipase activity induced by Bp-PLA_2_-TxI from *Bothrops pauloensis* snake venom. The indirect hemolysis assay was determined by measuring the hemolytic halo in mm^2^ after 24 h of incubation of 5 µg of BpPLA_2_-TXI previously incubated with different buffers (**A**) and temperatures (**B**). For all the experiments, data are expressed as means ± SEM (*n* = 3).

The indirect hemolytic activity was performed to assess the stability of BpPLA_2_-TXI. The protein was stable when submitted to different pH values (3.5, 5.2, 6.0, 7.5, 9.5, 10.5) (Figure 3A) or temperatures (4, 20, 25, 37, 45, 60 and 100 °C) (Figure 3B). However, PLA_2_ activity was reduced when BpPLA_2_-TXI was subjected to 100 °C for 30 min, suggesting that denaturation causes loss of protein stability, thereby decreasing its activity. The majority of PLA_2_s from snake venoms are highly stable due to the presence of intra-chain disulfide bonds present in their structures [8,13]. 

**Figure 4:**
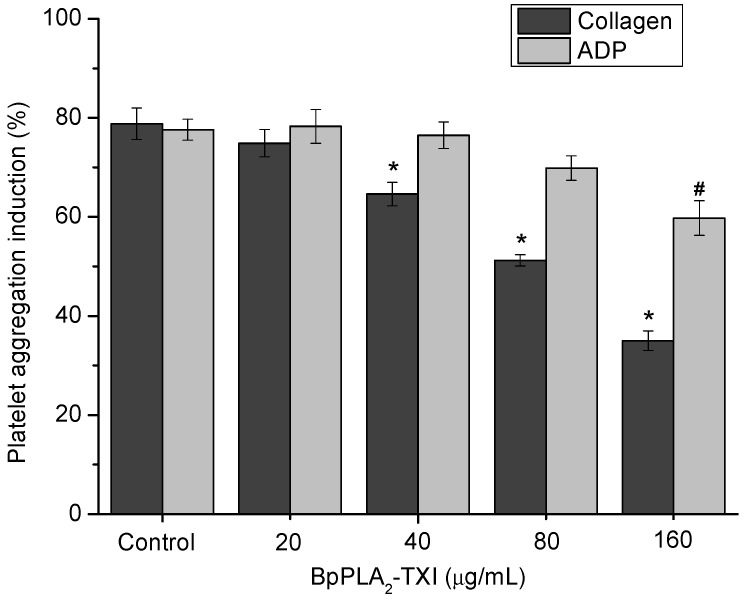
Platelet aggregation inhibition induced by BpPLA_2_-TXI. Different concentrations (20, 40, 80 and 160 µg/mL) of BpPLA_2_-TXI were preincubated at 37 °C for 10 min under stirring with washed platelets ((3–4) × 10^5^ cells/µL) and then platelet aggregation was initiated with ADP (20 µM) or collagen (10 µg/mL). Results are presented as percent of platelet aggregation (mean ± S.D., *n* = 3). Statistically significant results compared to collagen (*) or ADP (^#^) (*p* < 0.05) are shown.

BpPLA_2_-TXI inhibited platelet aggregation induced by collagen and ADP in a dose-dependent manner (Figure 4). Acidic PLA_2_s Asp-49 from several bothropic snake venoms are able to inhibit platelet aggregation induced by physiological agonists such as collagen and ADP [8,14]. The anti-platelet effect induced by PLA_2_s might be due to direct interference of the catalytic site in this activity or because of the C-terminal region of these proteins [8]. Some studies demonstrated that, when PLA_2_s are modified by bromophenacyl bromide (p-BPB), the His48 residue present in the active site is alkylated, resulting in a drastic loss or reduction of the enzymatic activity and/or pharmacological effects of PLA_2_s [8,15,16,17]. BpPLA_2_-TXI was efficient in inhibiting the platelet aggregation, although further experiments should be conducted to increase the knowledge of the mechanism of action of BpPLA_2_-TX on platelet aggregation.

**Figure 5:**
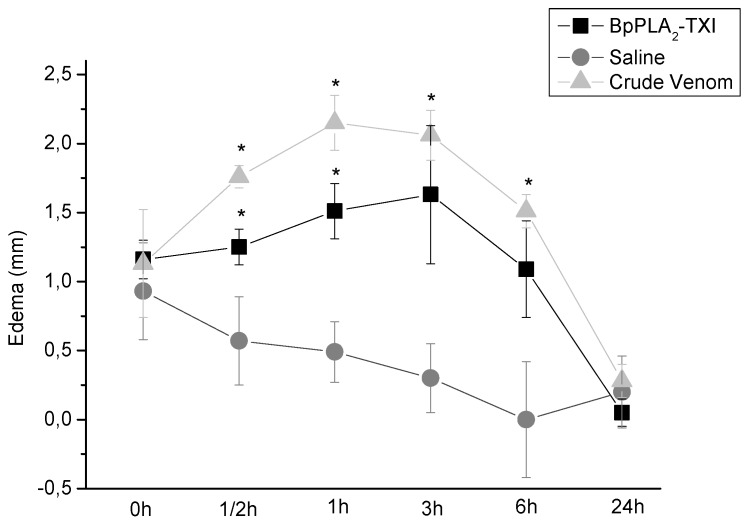
Edema-inducing activities of BpPLA_2_-TXI or crude venom. Paw edema in Swiss mice after injection of BpPLA_2_-TXI or crude venom (10 µg/50 µL Saline). Results are reported as mean ± S.D. (*n* = 3). (*) Statistically significant results compared to saline (*p* < 0.05) are shown.

BpPLA_2_-TXI also induced mouse paw edema (Figure 5), which was more extensive 3 h after administration of the toxin. Some D49 acidic phospholipase A_2_ forms have been characterized and have also shown similar effects [11,12,14]. Several factors may influence edema induction by these toxins that catalyze the breakdown of the phospholipids and release arachidonic acid, a precursor of eicosanoids, which can mediate the inflammatory response and induce edema [18]. 

**Figure 6:**
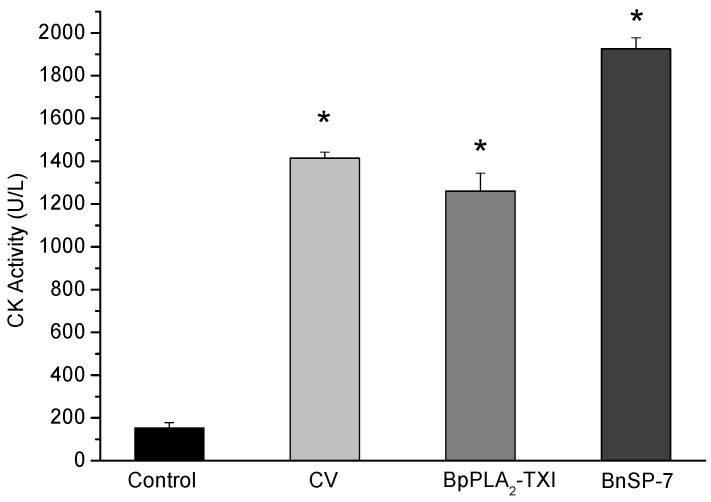
Myotoxic activity of BpPLA_2_-TXI in mice. Animals were injected i.m. with crude venom. Acidic BpPLA_2_-TXI or basic BnSP-7 (20 µg/50 µL Saline) and plasma CK activity was measured 3h after injection. Control mice were injected with only saline. Results are reported as mean ± S.D. (*n* = 3). (*) Statistically significant results compared to saline (*p* < 0.05) are shown.

BpPLA_2_-TXI induced significant increases in the plasma CK (creatine kinase) levels 3h after administration of the toxin in the gastrocnemius muscle. However, this effect was reduced when compared with CK levels induced by BnSP-7, a basic K49 PLA_2_ (Figure 6). Some D49 PLA_2_s do not present myotoxicity [12,19,20], but some studies have reported that these toxins may injure muscle fibers to a lesser degree [11,21].

The mechanism by which these toxins induced the myotoxicity is not precisely defined. Some works suggest an independent mechanism from the catalytic site, provoking a disturbance of the sarcolema through the insertion of the toxin in the C-terminal region inside the plasmatic membrane, leading to influx of calcium and consequent muscular necrosis [22]. However, other authors who have accomplished modification in the PLA_2_ with *p*-BPB have observed a loss or decrease of myotoxic activity [21], suggesting that myotoxicity of the acidic D49 PLA_2_ is directly related to the catalytic site. 

The cytotoxicity of the BpPLA_2_-TXI on some tumor cells was evaluated on MEF (Mouse Embryonic Fibroblast), Sarcoma 180 (TIB-66), TG-180 (CCRF S 180 II) and Ovarian Carcinoma (OVCAR-3). BpPLA_2_-TXI presented low cytotoxicity against TG-180 (CCRF S 180 II) and Ovarian Carcinoma (OVCAR-3) and did not show such activity against MEF (Mouse Embryonic Fibroblast) and Sarcoma 180 (TIB-66) (data not shown). Several authors have reported that some basic snake venom PLA_2_s produce high cytotoxic activity against tumor cells [23,24]. Meanwhile, other studies have revealed that the acidic PLA_2_ present low or no cytotoxicity [8,19]. 

**Figure 7:**
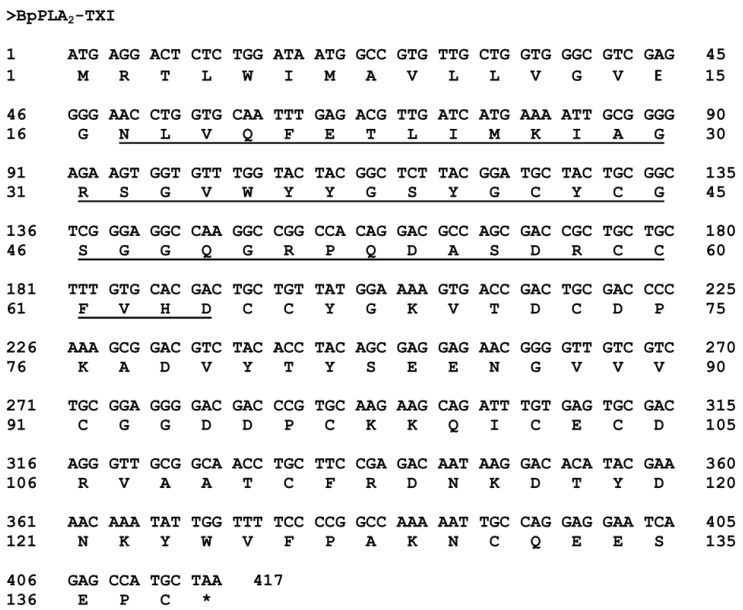
Complete sequence of the cDNA coding BpPLA_2_-TXI. The N-terminal sequence of the mature protein obtained by the Edman method is underlined. The initial region corresponds to the signal peptide. The symbol (*) represents the stop codon.

The sequence of the first 48 amino acid residues from BpPLA_2_-TXI was determined by Edman degradation (Figure 7) and the complete sequence was deduced further by cDNA. This sequence was recorded in the GenBank under Accession No. JK998826.

The nucleotide sequence of the BpPLA_2_-TXI showed 417 bp, codifying a mature protein of 122 amino acid residues (Figure 7). These results are in agreement with other works concerning acidic PLA_2_s, which presents 120–135 amino acid residues and 400–500 bp [9,19,20,25]. Analysis of the PLA_2_ primary sequence reveals that this enzyme possesses not only an Asp residue in the 49th position (D49) but also many other conserved residues (24YGCYCGWGG32; H48) involved at the Ca^2+^ binding and catalytic sites. 

**Figure 8:**
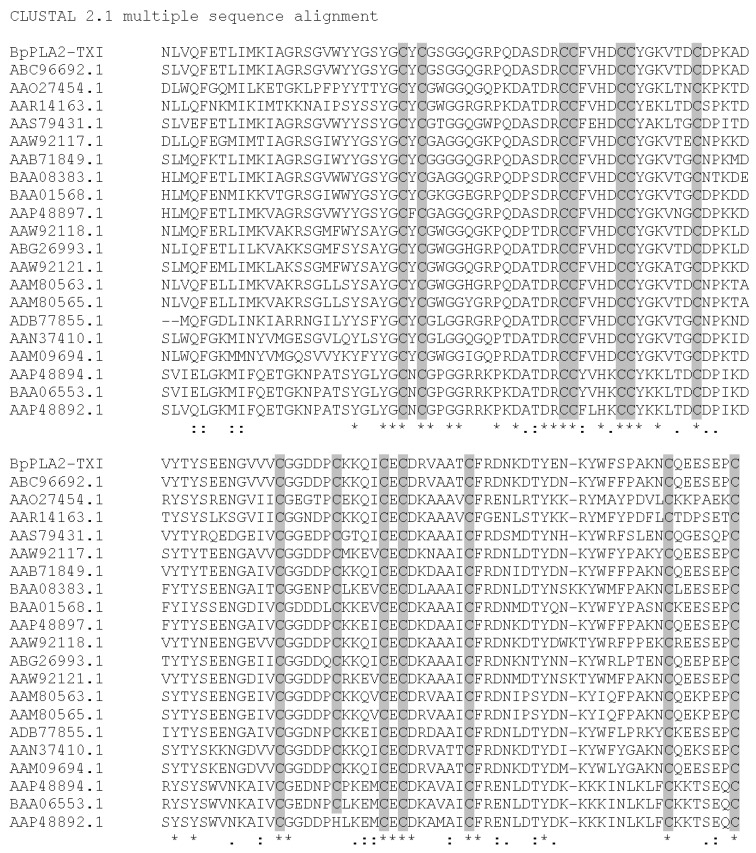
Multiple alignment of BpPLA_2_-TXI from *B. pauloensis* and the most similar PLA_2_ (acidic and basic; D49 and K49) sequences (by protein blast) from other snake venoms using ClustalW program. The cysteine residues are marked in grey. The symbol (*) represents conserved amino acid residues in all analyzed sequences. The symbol (**–**) represents gaps introduced in the sequences to maximize the alignment. The symbols (**:**) and (**.**) represent amino acid residues with same and different chemical characteristics, respectively. (ABC96692.1 *Bothropoides erythromelas*; AAO27454.1 and AAN37410.1 *Bothrops jararacussu*; AAR14163.1, ABG26993.1 and AAS79431.1 *Sistrurus catenatus*; AAM09694.1 *Bothropoides insularis*; AAW92118.1 *Cerrophidion godmani*; AAW92121.1 *Trimeresurus gracilis*; AAM80563.1 and AAM80565.1 *Crotalus viridis viridis*; BAA08383.1 *Ovophis okinavensis*; BAA01568.1 *Protobothrops flavoviridis*; AAP48897.1, AAP48894.1 and AAP48892.1 *Viridovipera stejnegeri*; AAW92117.1 *Bothriechis schlegelii*; AAB71849.1 *Gloydius halys*; ADB77855.1 *Lachesis muta*; BAA06553.1 *Trimeresurus gramineus*.

BpPLA_2_-TXI was aligned with other PLA_2_ sequences from *B. pauloensis* (results not shown). High identity was visualized (about 77%) with Bp-PLA_2_ [11], as expected, since both are acidic D49 PLA_2_s. Multiple alignments of the BpPLA_2_-TXI sequence with other acidic D49 PLA_2_s are shown in Figure 8. The best alignment scores pointed by ClustalW were with PLA_2_ from *Bothrops erythromelas* and *Sistrurus catenatus*, representing 99% and 89%, respectively. BpPLA_2_-TXI possesses many conserved domains that are common to D49 PLA_2_s, including 14 cystein residues which form seven disulfide bonds, and presents other residues involved with the calcium binding and catalytic sites as well. According to the results published by Rodrigues *et al.* [11] and of the present study, we suggest that Bp-PLA_2_ and BpPLA_2_-TXI may be isoforms present in *B. pauloensis* snake venom.

**Figure 9:**
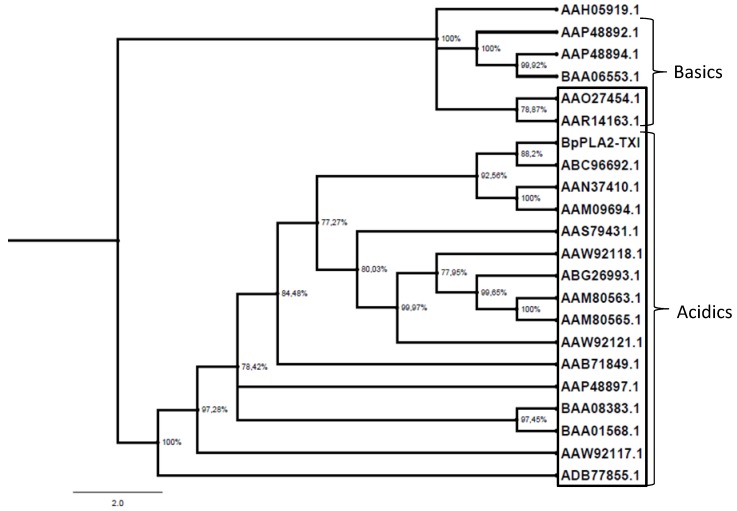
Evolutionary PLA_2_ relationships. The evolutionary history was inferred using the neighbor-joining method. Phylogenetic analyses were conducted by the program MrBayes. The PLA_2_s from snake venoms (deposited in GenBank) were grouped into two branches (acidic and basic); those shown inside the box are D49 PLA_2_ and those outside the box are K49. Human Synovial PLA_2_ (NCBI: AAH05919.1) sequence was included as outside group.

The PLA_2_ gene is involved in an accelerated evolution by substitutions in protein-encoding regions and appears to be universal for all the PLA_2_-encoding genes in snake venom glands of the crotaline snakes [26,27,28,29]. As shown in Figure 9, BpPLA_2_-TXI, together with other acidic PLA_2_s, formed a separate group from basic PLA_2_s, suggesting that the replacement of the D49 by the K49 residue is a derived characteristic, although additional studies need to be accomplished in order to elucidate this aspect. Tsai *et al.* [30], when isolating and characterizing several PLA_2_s from *Crotalus viridis viridis* snake venoms from different regions, showed that these acidic PLA_2_s were capable of inhibiting platelet aggregation and presented a glutamate (E) residue at Position 6. BpPLA_2_-TXI also posseses the E6 residue and is able to inhibit platelet aggregation. 

## 3. Experimental Section

### 3.1. Material and Animals

*B. pauloensis* dried crude venom was purchased from Serpentarium Bioagents (Batatais, SP, Brazil) and maintained at −20 °C. This serpentarium is registered with the Brazilian Institute of the Environment and Renewable Natural Resources (n 471301). Male Swiss mice (18–25 g) were obtained from and maintained in the Center for Animal Experimentation and Housing (CEBEA) at the Federal University of Uberlândia (UFU), Uberlândia, MG, Brazil. Animal experimentation procedures were approved by the UFU Ethics Committee for Animal Utilization (protocol number 046/09). CK-Nac Kit was purchased from Analisa (Belo Horizonte, Brazil). Adenosine diphosphate (ADP) and Collagen were acquired from Helena Laboratories (Beaumont, TX, USA). CM-Sepharose resin was purchased from Amersham Biotec (São Paulo, Brazil). All other reagents used were of analytical grade.

### 3.2. Isolation of Acidic Phospholipase A_2_

The acidic PLA_2_ was isolated by a combination of an ion exchange chromatography on CM-Sepharose fast flow [31], a gel filtration Sephacryl S-300 [32] and an ion exchange Capto-Q column (Amersham Biotec, São Paulo, Brazil), according to methods previously standardized by our research group. Initially, a sample containing 170 mg of desiccated *B*. *pauloensis* venom was dispersed into 2.0 mL of 0.05 M ammonium bicarbonate (AMBIC, Sigma, St. Louis, MO, USA) buffer, pH 7.8, cleared by centrifugation at 10,000 × *g* for 10 min at 4 °C and submitted to CM-Sepharose Fast Flow column (2.0 × 20 cm) which was previously equilibrated and initially eluted with the same buffer. A linear gradient was then applied up to 0.05 M Ambic buffer, pH 7.8 and fractions of 1 mL/tube were collected at a flow rate of 6.6 mL/h.

The fraction with PLA_2_ activity (CM1) was collected and gel filtrated on a Sephacryl S-300 HR HiPrep 26/60 (GE HealthCare, Uppsala, Sweden), equilibrated and eluted with 0.05 M Ambic buffer, pH 7.8. Fractions of 2.0 mL/tube were collected at a flow rate of 12.0 mL/h. The fraction with PLA_2_ activity (S4) was further submitted to an anionic exchange HiTrap Q FF column (GE HelthCare, Uppsala, Sweden) equilibrated with 0.05 M AMBIC buffer, pH 7.8. A linear gradient was then applied up to 0.5 M AMBIC buffer (AMBIC, Sigma, St. Louis, MO, USA), pH 7.8 and fractions of 1.0 mL/tube were collected at a flow rate of 6.0 mL/h. The active fraction (Q2) was rechromatographed by reverse-phase chromatography on a C2–C18 µRPC 4.6/100 (GE HelthCare, Uppsala, Sweden) column, previously equilibrated with solvent A (0.1% trifluoroacetic acid and 4% acetronitrile). The elution of the protein was conducted using a linear gradient of solvent B (0.1% trifluoroacetic acid and 80% acetronitrile) from 0% to 100%, at a flow rate of 0.5 mL/tube for 33 min. All steps of the purification procedure were carried out at room temperature (25 °C). The acidic PLA_2_ derived from RP-HPLC, named BpPLA_2_-TXI, was lyophilized and used for biochemical characterization and pharmacological studies.

### 3.3. Determination of *Mr*

Polyacrylamide gel electrophoresis (PAGE), in the presence of sodium dodecyl sulfate (SDS-PAGE), was performed according to Laemmli [33]. Samples were heated at 100 °C for 5 min and run under reducing (10% β-mercaptoethanol) and non-reducing conditions. The gel was stained with Coomassie Brilliant Blue R-250. The *Mr* was estimated by interpolation from a linear logarithmic plot of relative molecular mass *versus* migration distance. The molecular mass markers used were: bovine serum albumin (66 kDa), ovalbumin (45 kDa), glyceraldehyde-3-phosphate dehydrogenase (36 kDa), carbonic anhydrase (29 kDa), trypsinogen (24 kDa), trypsin inhibitor (20 kDa) and α-lactalbumin (14.2 kDa) (Amersham Biosciences, São Paulo, Brazil).

### 3.4. MALDI TOF Mass Spectrometry

BpPLA_2_-TXI was also submitted to mass spectrometric analysis, using an AutoFlex III MALDI-TOF mass spectrometer (Bruker Daltonics, Bremen, Germany) controlled by the software FlexControl 3.0 (Bruker Daltonics, Bremen, Germany). The sample was mixed with sinapinic acid matrix solution (1:1, *v*/*v*) directly onto a target plate (Bruker Daltonics, Bremen, Germany) and dried at room temperature. The mean mass of the protein was obtained in linear mode with external calibration, using Protein Calibration Standard (Bruker Daltonics, Bremen, Germany). The software Flex Analysis 3.0 (Bruker Daltonics, Bremen, Germany) was used for analysis of mass spectrometric data. 

### 3.5. Phospholipase A_2_ Activity

Phospholipase activity of BpPLA_2_-TXI was determined upon egg-yolk emulsion according to [34] and by the indirect hemolysis method, using washed mice erythrocytes and hen’s egg-yolk emulsion as substrate as described by [35]. 

BpPLA_2_-TXI stability at various pH intervals (50 mM ammonium formate pH 3.5; 50 mM sodium acetate pH 5.2; pH 6.0; 50 mM Tris HCl pH 7.5, 9.5 and 50 mM sodium borate pH 10.5) and temperatures (4 °C, 20 °C, 25 °C, 37 °C, 45 °C, 60 °C and 100 °C) was carried out as described by [35]. For this assay, 5 µg of BpPLA_2_-TXI were first incubated for 30 min with 50 μL of different buffer solutions at various pH intervals and temperatures, and the enzymatic assays were subsequently performed as described above [35].

### 3.6. Platelet Aggregation

The platelet aggregation was determined by using the method previously described by Fuly *et al.* [21], with modifications. Platelet-rich plasma (PRP) was prepared from mouse blood collected in the presence of citrate (0.31% *w*/*v*) and centrifuged at 360 × *g* for 15 min at room temperature. Washed platelets (WP) were prepared by centrifugation of PRP at 1800 × *g* for 12 min at room temperature. Platelets were resuspended in a Tyrode-BSA buffer and the final pH was adjusted to 7.5. Platelets were counted and their density adjusted to 3–4 × 10^5^ cells/µL. The platelet aggregation was measured turbidimetrically by AggRAM aggregometer (Helena Laboratories, Beaumont, TX, USA). WP suspended in Tyrode-BSA buffer (225 µL) were previously incubated at 37 °C for 2 min under stirring with 1 mM CaCl_2_ (final concentration). Aggregation was triggered with agonists after preincubation of platelets with different concentrations of PLA_2_ (20, 40, 80 and 160 µg/mL) at 37 °C for 30 min. Agonists ADP (20 µM) and collagen (10 µg/mL) (Helena Laboratories, Beaumont, TX, USA) were used as positive controls of reaction and their concentrations were determined according to the manufacturer’s instructions. 

### 3.7. Edema-Inducing Activity

Crude venom (10 µg/50 µL saline), BpPLA_2_-TXI (10 µg/50 µL saline) or saline were injected into the subplantar region of male Swiss mice (18–22 g, *n* = 5). After 0.5, 1, 2, 3, 6 and 24 h, paw edema was measured with the aid of a low-pressure spring caliper (Mitutoyo, Tokyo, Japan). Zero time values were then subtracted and the differences reported as mean ± S.D. 

### 3.8. Myotoxic Activity

Four groups of male Swiss mice (18–25 g, *n* = 4) were injected i.m. into the right *gastrocnemius* muscle with saline, crude venom (20 µg/50 µL Saline), BpPLA_2_-TXI (20 µg/50 µL Saline) or BnSP-7 (20 µg/50 µL), a Lys49 PLA_2_ previously isolated from *B. pauloensis* snake venom according to Soares *et al.* [18]. The control was 0.9% NaCl. After 3 h, the animals were anesthetized (ketamine^®^ 10% (0.05 mL/kg) + xylazine^®^ 2% (0.025 mL/kg)) and the blood was collected by cardiac puncture in heparin-coated tubes and centrifuged (2500 × *g* for 10 min at 4 °C) for separation of the serum. The amount of creatine kinase (CK) was then determined with 50 µL of serum, which was incubated for 3 min at 37 °C with 1.0 mL of the reagent according to the CK-Nac protocol (Gold Analisa Diagnóstica LTDA, Belo Horizonte, Brazil). Activity was expressed in U/L, with one unit resulting from the phosphorylation of 1 µmole of creatine/min at 25 °C. 

### 3.9. Cell Culture

MEF (Mouse Embryonic Fibroblast), Sarcoma 180 (TIB-66), TG-180 (CCRF S 180 II) and human ovarian carcinoma (OVCAR-3) cancer cell lines were maintained on RPMI 1640 medium supplemented with 2 mM l-glutamine, 1.5 g/L sodium bicarbonate, 4.5 g/L glucose, 10 mM HEPES, 1.0 mM sodium pyruvate, 10% fetal bovine serum and 10 μg/mL gentamicin. All cell culture reagents were purchased from Gibco (São Paulo, Brazil). All cell lines were maintained at 37 °C in 5% CO_2_ and 95% air containing more than 95% humidity.

### 3.10. Cytotoxicity Activity

Tumor cells were cultivated in appropriate flasks and continuously maintained at exponential growth. The tumor cells then were transferred to tubes and washed three times with RPMI medium at 500 × *g* for 10 min at room temperature. Cells were suspended in 5 mL of RPMI complete medium and dispersed in 96-well plates at a density of 1 × 10^5^ cells/well. After 24 h, the media were removed and fresh media, with or without different concentrations of BpPLA_2_-TXI (200–0.01 mg/mL), were added into the wells and incubated for 24 h. Cytotoxic activity of PLA_2_ was assayed with 3-(4,5-dimethylthiazol-2-yl)-2,5-diphenyltetrazolium bromide (MTT) staining and the MTT reduction was quantified at 540 nm. The cytotoxicity rate was calculated as follows: Cytotoxicity (%) = (1 − absorbance of the treated wells)/(absorbance of the control wells) × 100%.

### 3.11. Statistical Analysis

The results were presented as mean ± standard deviation (S.D.). Statistical significance of results was evaluated using the Student’s *t*-test. A value of *p* > 0.05 was considered significant.

### 3.12. Determination of Complete cDNA Sequence

The venom gland from *B. pauloensis* adult snake was dissected 3 days after venom extraction, when transcription was most stimulated [36]. The pair of venom glands was homogenized under liquid nitrogen and the total RNA was extracted using the Trizol (Gibco) method. tRNA was dissolved in sterile milli-Q water (Billerica, MA, USA) and submitted to reverse-transcription polymerase chain reaction (RT-PCR). Two microliters of this sample was used to amplify the specific PLA_2_ cDNA, by using the specific primer PLA_2_-forward (5'-GAT CAT GAA AAT TGC GGG GA-3'), which was designed according to the N-terminal sequence, as determined for BpPLA_2_-TXI, and the universal M13 reverse primer (5'-CAG GAA ACA GCT ATG AC-3' GE Healthcare, Uppsala, Sweden) by PCR. After the amplification of the obtained cDNA, the PCR products were analyzed in regard to the amplified size by means of gel electrophoresis on 1% agarose. The gel was stained with ethidium bromide (0.5 mg/mL) and revealed under UV light. The PCR bands were purified by using the Concert Rapid PCR Purification System (Gibco BRL, São Paulo, Brazil) kit, according to the manufacturer’s specifications. The amplified fragment was cloned into a p-GEM-T Easy Vector System (Promega^®^, São Paulo, Brazil). Bacteria were selected on a medium containing ampicillin and visualized after adding isopropylthio-β-galactoside (IPTG) and X-Gal in the culture medium. After selection, the colonies of recombinant bacteria were assayed by PCR and gel electrophoresis for cloning confirmation. The PCR products were purified and submitted to sequencing, using the kit DYEnamic ET Terminator Cycle Sequencing Kit (GE Healthcare Uppsala, Sweden) in a MEGA-BACE 1000 automated DNA sequencer (GE Healthcare, Uppsala, Sweden). The Base Caller Cimarron 3.12 software (Amersham Biosci., Sunnyvale, CA, USA) was used in order to analyze the electropherograms and generate the sequences. The cDNA sequences corresponding to acidic PLA_2_s were identified and the full-length sequence was obtained.

### 3.13. Phylogenetic Analysis

The predicted sequences of the acidic PLA_2_ (BpPLA_2_-TXI) and other snake venom PLA_2_s were aligned by ClustalW program (UCD, Dublin, Ireland; http://www.ebi.ac.uk/Tools/clustalw2/index.html) while employing Human Synovial PLA_2_ as the out-group. Datasets were analyzed using Bayesian inference implemented on MrBayes, version 3.2.1 [37] using lset rate *s* = invgamma with prset aamodelpr = mixed, which enables the program to optimize between nine different amino acid substitution matrices. The analysis was performed by running a minimum of 1.5 × 10^7^ generations in four chains, and saving every 100th tree. The log-likelihood score of each saved tree was plotted against the number of generations to establish the point at which the log likelihood scores reached their asymptote, and the posterior probabilities for clades established by constructing a majority-rule consensus tree for all trees generated after completion of the burn-in phase.

## 4. Conclusions

BpPLA_2_-TXI isolated from *B. pauloensis* snake venom is an acidic D49 PLA_2_ that shows high catalytic activity, platelet aggregation inhibition, edematogenic and myotoxic activities. This enzyme shares a high degree of sequence identity and a great similarity with other acidic PLA_2_s, especially with D49 and E6, thus forming a group distinct from the basic PLA_2_s. 
